# Individual-level agreement between coverage surveys and treatment registers in mass drug administration: A secondary analysis of the multi-country DeWorm3 cluster randomized trial

**DOI:** 10.1371/journal.pntd.0014740

**Published:** 2026-09-21

**Authors:** Elohor Oborevwori, Kristjana Ásbjörnsdóttir, Sean Galagan, Katherine Sharrock, Kumudha Aruldas, Saravanakumar Puthupalayam Kaliapan, Rachael Pullan, Robin Bailey, Khumbo Kalua, James Simwanza, Stefan Witek-McManus, Moudachirou Ibikounlé, Adrian J. F. Luty, Sitara Swarna Rao Ajjampur, Arianna Means, Judd L. Walson

**Affiliations:** 1 Department of Research, Johns Hopkins University School of Nursing, Baltimore, Maryland, United States of America; 2 Centre of Public Health Sciences, University of Iceland, Reykjavík, Iceland; 3 Department of Global Health, University of Washington, Seattle, Washington, United States of America; 4 The Wellcome Trust Research Laboratory, Division of Gastrointestinal Sciences, Christian Medical College, Vellore, India; 5 Department of Disease Control, Faculty of Infectious and Tropical Diseases, London School of Hygiene and Tropical Medicine, London, United Kingdom; 6 Blantyre Institute for Community Outreach, Lions Sight First Eye Hospital, Blantyre, Malawi; 7 Clinical Research Department, Faculty of Infectious and Tropical Diseases, London School of Hygiene and Tropical Medicine, London, United Kingdom; 8 School of Population and Public Health, University of British Columbia, Vancouver, British Columbia, Canada; 9 Institut de Recherche Clinique du Benin, Abomey-Calavi, Benin; 10 Centre de Recherche pour la lutte contre les Maladies Infectieuses Tropicales, Université d’Abomey-Calavi, Abomey-Calavi, Benin; 11 Université Paris Cité, Institute of Research for Development, (IRD), National Institute of Health and Medical Research (INSERM), International Health of Mother and Child (MERIT), Paris, France; 12 Departments of International Health, Medicine and Pediatrics, Johns Hopkins University, Baltimore, Maryland, United States of America; University Hospital Bonn: Universitatsklinikum Bonn, GERMANY

## Abstract

**Background:**

Accurate measurement of treatment coverage is critical for the monitoring and evaluation of mass drug administration (MDA) programs, yet post-treatment coverage surveys are subject to multiple sources of bias and may not accurately reflect true individual-level treatment coverage. Using data from the DeWorm3 study, a multi-country randomized trial assessing the feasibility of interrupting soil-transmitted helminth (STH) transmission, we compared coverage estimates from treatment registers with post-treatment coverage surveys and identified factors associated with discordant treatment reporting.

**Methods:**

The DeWorm3 trial analyzed individual-level treatment data across six rounds of biannual MDA with albendazole for STH in Benin, India, and Malawi (2018–2020). Coverage surveys and treatment registers were linked using unique identifiers. This analytic sample included 12,162 individuals with matched survey and register records and non-missing treatment status from the final round of the intervention-arm. Concordance was summarized using percent discrepancy and Cohen’s kappa, and site-specific logistic regression models assessed factors associated with discrepancy.

**Results:**

While aggregate coverage was high (>95%) across all sites, individual-level discordance at round six occurred in 6.12% of individuals in Benin, 2.00% in India, and 4.39% in Malawi, driven predominantly by higher reporting in coverage surveys relative to register records. Kappa values were interpreted descriptively as treatment coverage was very high. Periurban/urban residence was associated with higher odds of discrepancy in Benin (OR: 3.65, 95% CI: 2.56–5.28) and Malawi (OR: 1.54, 95% CI: 1.11–2.12). In Malawi, male sex was also associated with higher discrepancy, while MDA awareness was associated with lower discrepancy. No covariates were significantly associated with discrepancy in India.

**Conclusions:**

Individual-level discordance between coverage surveys and treatment registers was present across all sites and rounds, with surveys consistently estimating higher levels of treatment. Predictors varied by site, suggesting measurement error is driven by site-level programmatic and contextual factors rather than individual demographic characteristics. Programs should implement systematic validation approaches as high aggregate coverage estimates may mask meaningful individual-level measurement error.

## Author summary

When large-scale medicine distribution programs treat entire communities for infectious diseases, knowing how many people actually received treatment is critical. Programs typically measure this by asking people whether they took their medication in a household survey conducted shortly after treatment. In this study, using data from the DeWorm3 trial, a large randomized trial conducted in Benin, India, and Malawi, we directly compared what people reported in surveys with what was recorded at the time of treatment for the same individuals. We found that while overall treatment rates appeared high in both data sources, a meaningful proportion of individuals gave responses in surveys that did not match their treatment records, with surveys generally reporting higher treatment than the records suggested. Importantly, the factors that predicted these mismatches differed substantially across the three countries, suggesting that measurement inaccuracies are shaped by local context rather than any universal characteristic of the individuals surveyed. These findings highlight that high overall treatment rates can mask important inaccuracies at the individual level, and that programs sole reliance on surveys to assess whether treatment goals are being met may be problematic, particularly when used to make critical programmatic decisions.

## Introduction

Mass drug administration (MDA) is a cornerstone strategy for controlling several preventive chemotherapy neglected tropical diseases (PC-NTDs), including lymphatic filariasis, onchocerciasis, schistosomiasis, and soil-transmitted helminth (STH) infections. These programs aim to achieve minimum coverage thresholds to ensure population level impact. The WHO recommends at least 75% of eligible populations receive treatment annually, as high coverage is necessary to reduce disease burden and associated morbidity [1]. Accurate coverage measurement is therefore critical as a process indicator for programmatic success, for determining efficient resource use and for identifying opportunities to improve implementation.

Current coverage measurement relies on two primary approaches; 1) administrative treatment registers documenting medication distribution during campaigns and 2) post-MDA coverage surveys capturing self-reported treatment amongst a subset of the treated population, typically 2–12 weeks following an MDA campaign [2]. These methods frequently produce discrepant results, with prior studies reporting inconsistent patterns in the direction of discrepancy across settings, including both overestimation and underestimation of coverage by program records relative to survey estimates, likely reflecting variation in data quality, population denominator accuracy, and local implementation factors [3,4]. This measurement inconsistency creates uncertainty about whether programs are achieving critical coverage thresholds and limits the ability to identify factors that influence treatment uptake across different contexts.

Individual-level agreement is important because similar aggregate coverage estimates can mask discordant classification of who was or was not treated. This may limit the ability to identify missed groups, evaluate predictors of treatment uptake, and interpret coverage estimates in settings close to programmatic thresholds. The DeWorm3 trial provides a unique opportunity to examine this question because coverage survey responses can be linked to treatment registers collected by well-trained study staff employing systematic, household-level data collection procedures across multiple rounds of community-wide MDA.

In this secondary analysis, we quantified the magnitude and direction of individual-level disagreement between coverage surveys and trial-based treatment registers across six rounds of community-wide MDA in the multi-country DeWorm3 trial, conducted in Benin, India, and Malawi between 2018–2020 [5,6]. We also identified demographic and contextual factors associated with discrepant treatment classification. These findings provide evidence-based guidance for improving coverage measurement accuracy and developing context-adapted implementation strategies for STH control and other disease control programs.

## Results

The merged dataset for MDA round 6 comprised 24,751 individuals successfully linked between coverage survey and treatment register datasets across the three study sites (8,464 in Benin, 7,223 in India, and 9,064 in Malawi). After restricting the sample to individuals in the intervention arm with non-missing treatment status in both data sources, the final round six analytic sample included 12,162 individuals: 4,069 in Benin, 3,747 in India, and 4,346 in Malawi (S1 Fig). Characteristics of individuals included versus excluded from the round 6 intervention-arm analytic sample are presented in S1 Table.Table 1 presents the demographic characteristics of the analytic sample; complete baseline characteristics of the DeWorm3 trial population are reported in the primary trial publication [7].

**Table 1:** Demographic characteristics of the analytic sampled study population in Benin, India and Malawi.

Characteristics	Benin (N = 4069)^1^	India (N = 3747)^1^	Malawi (N = 4346)^1^	Total (N = 12162)^1^	p-value^2^
**Age category, n (%)**					<0.001
**≤ 1 year**	0 (0%)	0 (0%)	0 (0%)	0 (0%)	
**1–4 years**	403 (9.9%)	190 (5.1%)	502 (11.6%)	1095 (9%)	
**5–14 years**	1246 (30.6%)	556 (14.8%)	1547 (35.6%)	3349 (27.5%)	
**≥ 15 years**	2420 (59.5%)	3001 (80.1%)	2296 (52.8%)	7717 (63.5%)	
**Unknown**	0 (0%)	0 (0%)	1 (0%)	1 (0%)	
**Sex, n (%)**					0.048
**Male**	1894 (46.5%)	1835 (49%)	2024 (46.6%)	5753 (47.3%)	
**Female**	2175 (53.5%)	1912 (51%)	2322 (53.4%)	6409 (52.7%)	
**Religion, n (%)** ^**a**^					<0.001
**Hinduism**	0 (0%)	3635 (97%)	0 (0%)	3635 (29.9%)	
**Christianity**	2593 (63.7%)	51 (1.4%)	193 (4.4%)	2837 (23.3%)	
**Islam/Muslim**	83 (2%)	61 (1.6%)	4150 (95.5%)	4294 (35.3%)	
**Voodoo**	1036 (25.5%)	0 (0%)	0 (0%)	1036 (8.5%)	
**None**	191 (4.7%)	0 (0%)	0 (0%)	191 (1.6%)	
**Unknown**	166 (4.1%)	0 (0%)	3 (0.1%)	169 (1.4%)	
**Education, n (%)**					<0.001
**No education**	1025 (25.2%)	849 (22.7%)	1132 (26%)	3006 (24.7%)	
**Any Primary education**	435 (10.7%)	360 (9.6%)	769 (17.7%)	1564 (12.9%)	
**Any Middle education**	0 (0%)	451 (12%)	0 (0%)	451 (3.7%)	
**Any Secondary education**	318 (7.8%)	519 (13.9%)	126 (2.9%)	963 (7.9%)	
**Any Higher secondary education**	115 (2.8%)	194 (5.2%)	0 (0%)	309 (2.5%)	
**College/Diploma/University**	99 (2.4%)	280 (7.5%)	0 (0%)	379 (3.1%)	
**Unknown**	2077 (51%)	1094 (29.2%)	2319 (53.4%)	5490 (45.1%)	
**Marital status, n (%)**					<0.001
**Unmarried**	664 (16.3%)	463 (12.4%)	420 (9.7%)	1547 (12.7%)	
**Married monogamous**	1170 (28.8%)	1927 (51.4%)	1172 (27%)	4269 (35.1%)	
**Married polygamous**	63 (1.5%)	0 (0%)	138 (3.2%)	201 (1.7%)	
**Separated**	41 (1%)	21 (0.6%)	125 (2.9%)	187 (1.5%)	
**Divorced (Legally)**	0 (0%)	1 (0%)	33 (0.8%)	34 (0.3%)	
**Widow/Widower**	104 (2.6%)	271 (7.2%)	111 (2.6%)	486 (4%)	
**Unknown**	2027 (49.8%)	1064 (28.4%)	2347 (54%)	5438 (44.7%)	
**Wealth index quintile, n (%)** ^**b**^					0.001
**Lowest quintile**	705 (17.3%)	626 (16.7%)	755 (17.4%)	2086 (17.2%)	
**Low quintile**	667 (16.4%)	731 (19.5%)	765 (17.6%)	2163 (17.8%)	
**Medium quintile**	821 (20.2%)	710 (18.9%)	904 (20.8%)	2435 (20%)	
**High quintile**	922 (22.7%)	794 (21.2%)	864 (19.9%)	2580 (21.2%)	
**Highest quintile**	954 (23.4%)	886 (23.6%)	1055 (24.3%)	2895 (23.8%)	
**Unknown**	0 (0%)	0 (0%)	3 (0.1%)	3 (0%)	
**Cluster type, n (%)**					<0.001
**Rural**	1725 (42.4%)	2974 (79.4%)	3256 (74.9%)	7955 (65.4%)	
**Periurban/Urban**	2344 (57.6%)	773 (20.6%)	1090 (25.1%)	4207 (34.6%)	
**MDA awareness, n (%)**					<0.001
**Not aware**	696 (17.1%)	1427 (38.1%)	1711 (39.4%)	3834 (31.5%)	
**Aware**	3336 (82%)	1730 (46.2%)	2474 (56.9%)	7540 (62%)	
**Unknown**	37 (0.9%)	590 (15.7%)	161 (3.7%)	788 (6.5%)	

^a^Majority religion in the study area: Christianity in Benin; Islam in Malawi; Hinduism in India. Minority religions: Islam, Voodoo, or traditional religion in Benin; Christianity or other in Malawi; Christianity, Islam, or other in India.

^b^Wealth Index quintile calculated using Principal Components Analysis, taking the first principal component, on a list of household assets including house characteristics and appliance, technology, and livestock ownership (with additional fertilizer and water, sanitation and hygiene [WASH] access variables in Malawi only).

^1^n (%), ^2^Pearson’s Chi-squared test.

Notable demographic differences were observed between sites: India had a higher proportion of adults aged ≥15 years (80.1%) as compared to Benin (50.5%) and Malawi (52.8%). Malawi had the highest proportion of children under five years (11.46%) compared to Benin (9.9%) and India (5.1%). Sex distribution was relatively balanced across all sites, with females comprising 53.5% of the population in Benin, 51.0% in India, and 53.4% in Malawi.

Educational attainment varied substantially across sites, although these distributions should be interpreted cautiously because education was missing or not recorded for a substantial proportion of individuals. India had the highest proportion of individuals with college or university education (7.5%) as compared to Benin (2.4%) or Malawi (0%). Religious affiliation also differed markedly, with Hinduism predominant in India (97.0%), Islam predominant in the Malawi site (95.5%), and Christianity (63.7%) and Voodoo (25.5%) in Benin.

Wealth index quintiles showed relatively even distribution across all sites, as expected given that these were quintiles constructed using baseline census data in the same population. The highest income quintile represented 23.4% of the analytic sample in Benin, 23.6% in India, and 24.3% in Malawi, while the lowest income quintile represented 17.3%, 16.7%, and 17.4% respectively.

Cluster type also differed substantially across sites. Benin had the highest proportion of individuals residing in periurban or urban clusters at 57.6%, compared with 20.6% in India and 25.1% in Malawi. In contrast, rural clusters accounted for most of the analytic sample in India and Malawi, representing 79.4% and 74.9% of individuals, respectively, compared with 42.4% in Benin. Awareness of the MDA campaign before implementation was highest in Benin at 82%, compared with 56.9% in Malawi and 46.2% in India. The proportion with unknown awareness status was highest in India at 15.7%, compared with 3.7% in Malawi and 0.9% in Benin.

Concordance between coverage surveys and treatment registers was assessed descriptively across all six MDA rounds among matched individuals in the intervention arm, with round-specific sample sizes varying by site and MDA round (Table 2). Coverage estimates differed substantially between the two measurement methods across all three study sites (Table 2). Treatment coverage as assessed by coverage survey, representing the proportion of individuals who reported swallowing albendazole in post-MDA coverage surveys, was notably high across all sites: 98.21% in Benin, 99.07% in India, and 99.75% in Malawi. The proportion recorded as treated in treatment registers (log coverage), was 95.48% in Benin, 98.29% in India, and 95.81% in Malawi. The absolute difference between survey and log coverage estimates was 2.73 percentage points in Benin, 0.78 percentage points in India, and 3.93 percentage points in Malawi, with survey coverage consistently exceeding log coverage across all three sites.

**Table 2:** Coverage estimates, discrepancies, and reporting errors from coverage surveys and treatment registers by site and MDA round.

Site	Round	Sample (n)	Coverage survey (%)	Treatment log (%)	Difference (%)	Discrepancy (%)	Over reporting (%)	Under reporting (%)	kappa
**Benin**	1	3523	97.62	91.91	5.71	9.74	7.72	2.02	0.03
**Benin**	2	3679	97.91	95.24	2.66	5.65	4.16	1.49	0.15
**Benin**	3	3903	99.77	97.92	1.84	2.20	2.02	0.18	0.04
**Benin**	4	3425	98.74	97.17	1.58	3.74	2.66	1.08	0.07
**Benin**	5	3694	99.35	94.67	4.68	5.72	5.20	0.51	0.03
**Benin**	6	4069	98.21	95.48	2.73	6.12	4.42	1.70	0.01
**India**	1	3232	96.63	96.84	-0.22	5.97	2.88	3.09	0.05
**India**	2	3637	98.76	96.65	2.12	3.82	2.97	0.85	0.15
**India**	3	3554	99.35	98.85	0.51	1.63	1.07	0.56	0.09
**India**	4	3591	99.05	99.28	-0.22	1.34	0.56	0.78	0.19
**India**	5	3394	97.76	92.07	5.69	8.28	6.98	1.29	0.16
**India**	6	3747	99.07	98.29	0.78	2.00	1.39	0.61	0.23
**Malawi**	1	3582	99.58	93.86	5.72	6.45	6.09	0.36	0.01
**Malawi**	2	3562	99.89	93.77	6.12	6.29	6.20	0.08	0.01
**Malawi**	3	3704	99.38	95.25	4.13	5.10	4.62	0.49	0.04
**Malawi**	4	3845	99.56	95.99	3.56	4.19	3.88	0.31	0.05
**Malawi**	5	4091	99.12	96.70	2.42	3.64	3.03	0.61	0.12
**Malawi**	6	4346	99.75	95.81	3.93	4.39	4.16	0.23	0.01

Survey coverage indicates the percentage of individuals reporting swallowing albendazole in post-MDA coverage surveys. Log coverage indicates the percentage recorded as treated in treatment registers. Coverage difference represents the absolute difference between survey and log coverage estimates. Discrepancy indicates the percentage of individuals for whom treatment status differed between the two data sources. Over-reporting indicates individuals reporting treatment in the coverage survey but not recorded as treated in treatment registers; under-reporting indicates the converse.

Individual-level discrepancies, defined as cases where treatment status differed between the two data sources, occurred in 6.12% of individuals in Benin, 2.00% in India, and 4.39% in Malawi (Fig 1A). The direction of these discrepancies varied by site, though over-reporting (where individuals reported treatment in the coverage survey but were not recorded as treated in treatment registers) predominated in all settings, occurring in 4.42% of the sample in Benin, 1.39% in India, and 4.16% in Malawi. Under-reporting, where individuals did not report treatment in the coverage survey but were recorded as treated in treatment registers, occurred in 1.70% of individuals in Benin, 0.61% in India, and 0.23% in Malawi. These patterns indicate that coverage surveys tended to overestimate treatment receipt relative to treatment registers across all sites, with the magnitude of over-reporting varying substantially by setting.

**Fig 1:**
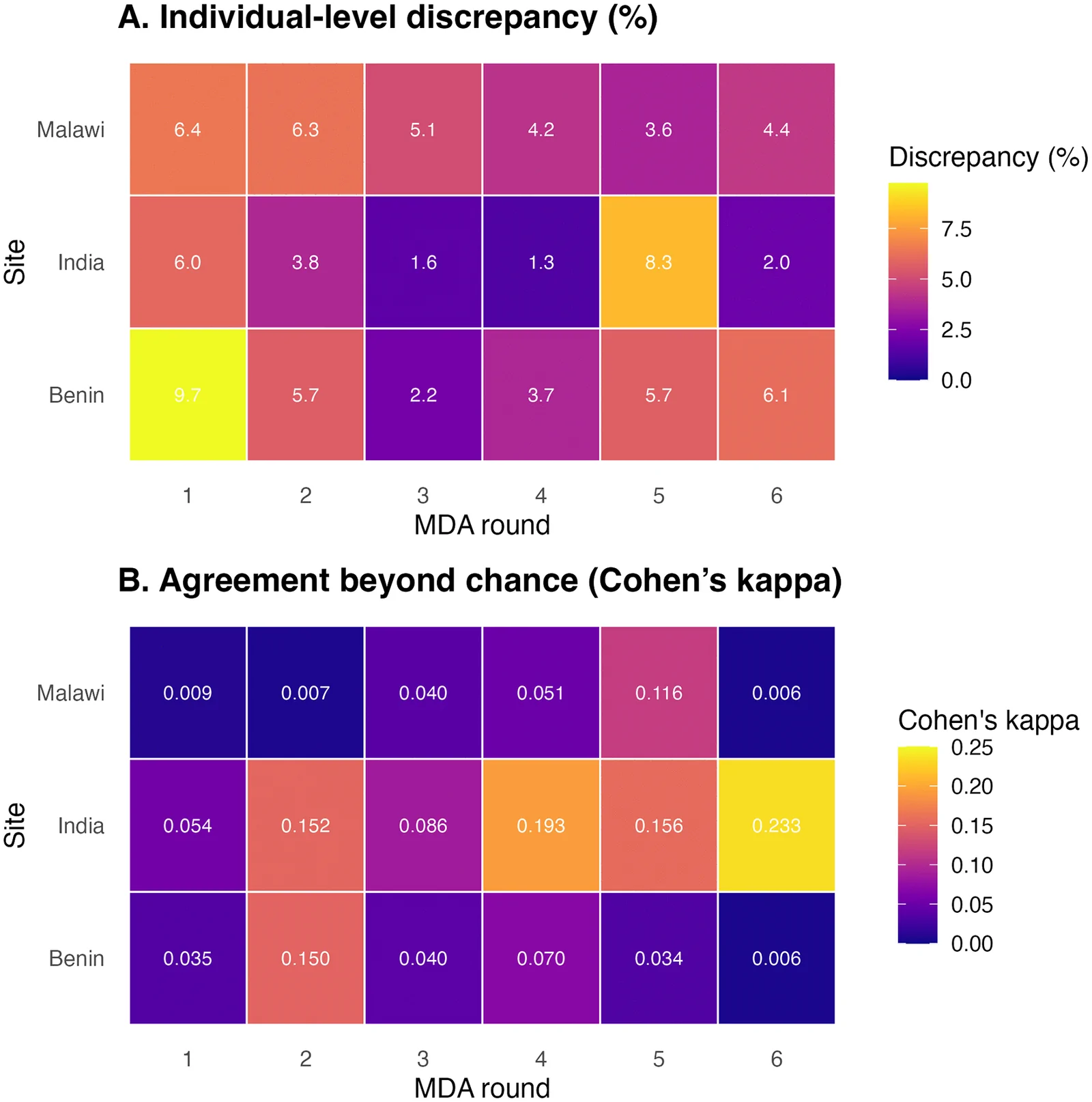
Discrepancies and agreement between coverage surveys and treatment registers across MDA rounds and sites.

Agreement between coverage surveys and treatment registers, assessed using Cohen’s kappa statistics, revealed poor concordance across all three study sites and throughout the six MDA rounds (Table 2, Fig 1B).

Comparison of agreement patterns across all six MDA rounds revealed consistently low kappa values throughout the study period. In Benin, kappa values ranged from 0.006 to 0.150 across rounds, with the highest value observed in round 2 (kappa = 0.15) and lowest in round 6 (kappa = 0.006). India demonstrated highest kappa values overall, ranging from 0.054 to 0.233, with the highest observed value of 0.233 in round 6. Malawi showed consistently low kappa values ranging from 0.006 to 0.116, with most rounds below 0.05.

Individual-level discrepancies varied across rounds but remained substantial throughout the study period (Fig 1A). In Benin, discrepancy rates ranged from 2.2% (round 3) to 9.7% (round 1), demonstrating considerable variability across rounds. India showed discrepancies ranging from 1.3% (round 4) to 8.3% (round 5), with a notable spike in round 5. Malawi exhibited the most stable pattern, with discrepancies ranging from 3.6% (round 5) to 6.4% (round 1). Overall, these findings show that high aggregate coverage estimates can coexist with individual-level disagreement between coverage surveys and treatment registers, and that the magnitude and direction of disagreement varied across sites and MDA rounds.

In the primary site-specific logistic regression models, education was excluded because it was missing or not recorded for a substantial proportion of individuals. We identified distinct patterns of factors associated with discrepancies between coverage surveys and treatment registers across the three study sites (Table 3, Fig 2).

**Table 3:** Site-specific logistic regression models of individual-level discrepancies between coverage surveys and treatment registers.

	Benin (n = 4,031)			India (n = 3,157)			Malawi (n = 4,179)		
Covariate	OR	95% CI	p-value	OR	95% CI	p-value	OR	95% CI	p-value
**Age (per 10-year increase)**	0.97	0.90, 1.04	0.5	1.07	0.93, 1.23	0.3	1.02	0.95, 1.10	0.6
**Sex**									
Female (ref)	1.0	1.0		1.0	1.0		1.0	1.0	
Male	0.93	0.71, 1.21	0.6	0.84	0.48, 1.46	0.5	1.37	1.02, 1.84	0.040
**MDA aware**									
Not aware (ref)	1.0	1.0		1.0	1.0		1.0	1.0	
Aware	0.92	0.67, 1.29	0.6	0.77	0.44, 1.34	0.3	0.66	0.49, 0.89	0.01
**Cluster type**									
Rural (ref)	1.0	1.0		1.0	1.0		1.0	1.0	
Periurban/Urban	3.65	2.56, 5.28	<0.001	1.29	0.66, 2.40	0.4	1.54	1.11, 2.12	0.01
**Minority religion**									
Majority religion (ref)	1.0	1.0		1.0	1.0		1.0	1.0	
Minority religion	0.80	0.58, 1.11	0.2	0.57	0.03, 2.66	0.6	1.16	0.59, 2.08	0.7
**Wealth index quintile**									
Lowest quintile (ref)	1.0	1.0		1.0	1.0		1.0	1.0	
Low quintile	1.07	0.68, 1.70	0.8	1.04	0.38, 2.93	>0.9	0.91	0.55, 1.52	0.7
Medium quintile	0.67	0.43, 0.43	0.09	0.68	0.22, 2.08	0.5	0.48	0.27, 0.85	0.01
High quintile	0.64	0.41, 1.01	0.52	1.66	0.69, 4.42	0.3	1.01	0.62, 1.65	>0.9
Highest quintile	0.58	0.38, 0.92	0.02	1.28	0.52, 3.44	0.6	1.26	0.81, 2.00	0.3

Abbreviations: CI = Confidence Interval, OR = Odds Ratio.

**Fig 2:**
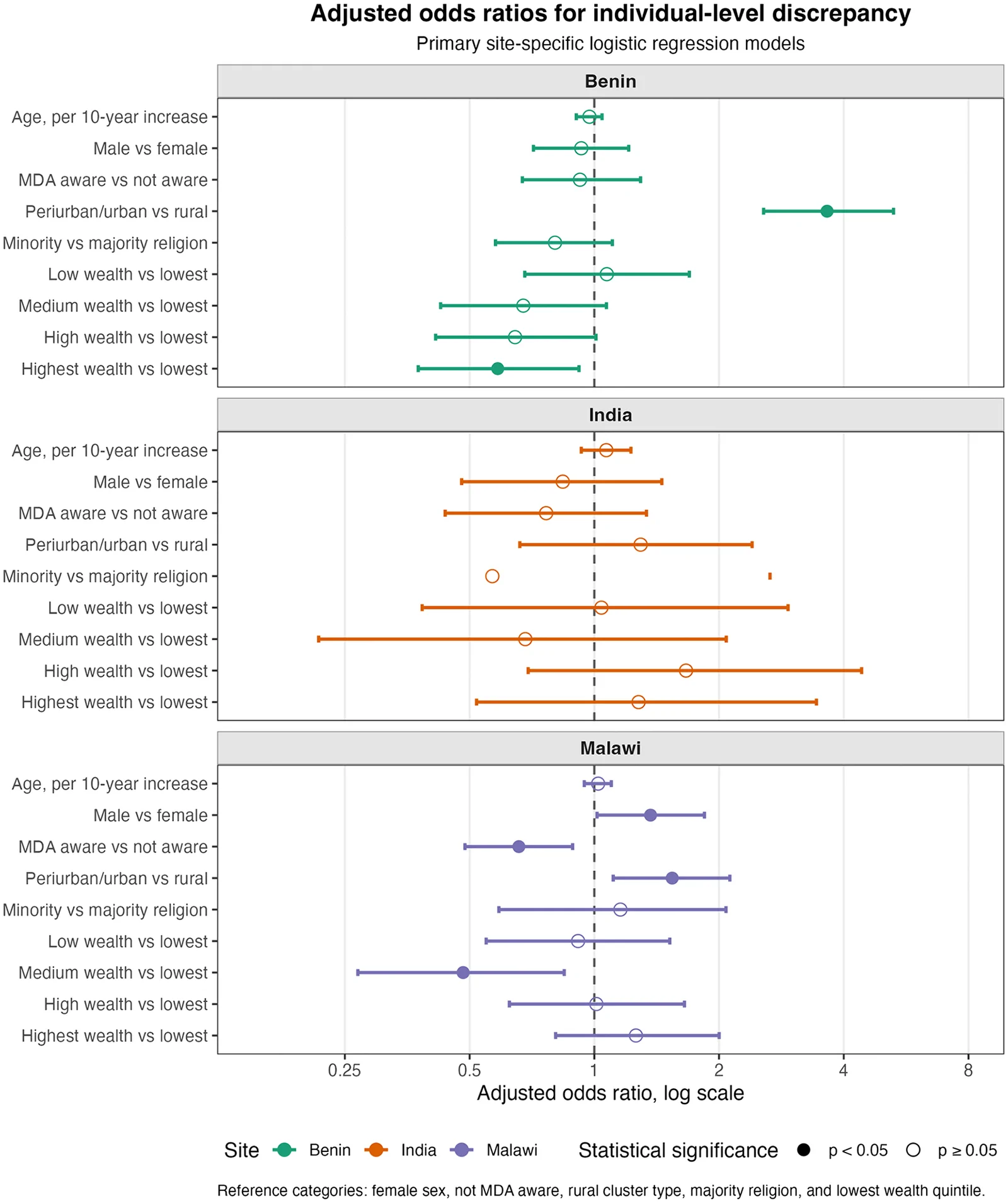
Site-specific adjusted odds ratios for predictors of discrepancy between self-reported and recorded MDA treatment.

In Benin, rural or periurban/urban cluster residence was associated with higher odds of discrepant treatment status compared to those in rural residence (OR: 3.65, 95% CI: 2.56-5.28, p < 0.001). Relative wealth was also associated with discrepancy: individuals in the highest income quintile had lower odds of discrepancy compared the lowest wealth quintile (OR: 0.58, 95% CI: 0.38–0.92, p = 0.018) compared with those in the lowest quintile (OR: 0.58, 95% CI: 0.38-0.92, p = 0.018). A similar pattern was observed for the high wealth quintile, although this did not reach statistical significance (OR: 0.64, 95% CI: 0.41–1.01, p = 0.052). Age, sex, MDA awareness, religion, and other wealth category levels were not significantly associated with discrepancy in Benin.

In India, none of the examined covariates were significantly associated with discrepancy. Age, sex, MDA awareness, cluster type, religion, and wealth quintile all had confidence intervals that included the null value. The lack of significant associations in India suggest that discrepancies in this setting may be more random rather than systematically associated with measured individual characteristics.

In Malawi, several covariates were significantly associated with discrepancy. Males had higher odds of discrepant treatment status compared with females (OR: 1.37, 95% CI: 1.02-1.84, p = 0.040). Individuals who were aware of the MDA campaign had lower odds of discrepancy compared with those who were not aware (OR: 0.66, 95% CI: 0.49–0.89, p = 0.006). Periurban or urban cluster residence was associated with higher odds of discrepancy compared with rural residence (OR: 1.54, 95% CI: 1.11–2.12, p = 0.009). Individuals in the medium income quintile had lower odds of discrepancy compared to those in the lowest income quintile (OR: 0.48, 95% CI: 0.27-0.85, p = 0.012) in Malawi. Age, religion and the remaining wealth quintiles were not significantly associated with discrepancy in Malawi.

As a sensitivity analysis (S2 Table), site-specific models were repeated including education as a categorical covariate. These education-included models retained a smaller analytic sample and were therefore interpreted cautiously. In Benin, periurban or urban cluster residence remained associated with higher odds of discrepancy, while the association between the highest wealth quintile and lower discrepancy was attenuated and no longer statistically significant. In India, no covariates were significantly associated with discrepancy in the education-included model, although the highest education category showed a non-significant trend toward higher odds of discrepancy. In Malawi, the education-included model showed some differences from the primary model: older age, male sex, and medium wealth were associated with lower odds of discrepancy, while selected education categories were associated with higher odds of discrepancy. However, because the education-included models excluded a substantial proportion of the analytic sample, education-related findings were considered exploratory.

## Discussion

Prior evaluations of MDA coverage have largely focused on comparing aggregate program-reported coverage with post-MDA survey estimates, often finding that administrative or program records overestimate coverage because of denominator inaccuracies, incomplete population enumeration, duplicate reporting, or routine recording errors [8–10]. Far fewer studies have examined whether the same individuals are classified consistently across data sources. This study extends the existing evidence by linking individual-level coverage survey responses to treatment register records across six rounds of community-wide albendazole MDA in three countries. Despite high overall coverage estimates, substantial individual-level discordance existed between coverage surveys and treatment registers and was present across all sites and rounds. The level of individual-level discordance observed in this study, while modest in absolute terms, indicates that similar aggregate coverage estimates can mask disagreement in who is classified as treated or untreated.

The predominance of higher reporting of treatment during coverage surveys in this study contrasts with previous studies where treatment registers typically overestimated coverage [8–10]. This reversal likely reflects the rigorous data collection procedures in DeWorm3, where trained field workers maintained prospective, census-linked treatment registers that were updated through repeated enumeration activities, likely reducing common sources of register overestimation such as inaccurate denominators, duplicate records, and aggregate tallying errors [11]. Also, coverage surveys relied on self-report or proxy report after MDA and may have been influenced by recall bias, social desirability bias, or confusion between treatment rounds [12]. The two data sources measured related but not identical constructs: surveys captured reported swallowing of albendazole, whereas treatment registers documented per-protocol treatment delivery, including instances where drugs were left at the household for absent individuals [13]. In routine settings with less rigorous administrative data, the balance between survey over-reporting and log overestimation may differ considerably.

The heterogeneity across sites suggests that measurement error may not be explained by shared demographic characteristics across settings. The association between discrepancy and urban setting in Benin suggests specific implementation or measurement challenges that may exist in densely populated settings. The absence of significant associations in India may suggest more optimal implementation quality or survey procedures. Finally, the demographic patterns associated with discrepancy in Malawi may indicate that treatment-seeking behaviours or reporting may vary systematically across subgroups in culturally specific ways.

These patterns indicate that measurement challenges arise from local implementation factors and health system characteristics, rather than universal phenomena. For example, the association between periurban or urban residence and higher discrepancy in Benin and Malawi may reflect challenges related to household availability, population mobility, treatment tracking, or survey follow-up in more densely populated areas. The association between MDA awareness and lower discrepancy in Malawi may indicate that individuals who were aware of the campaign had clearer recall or more consistent engagement with MDA activities. Because predictors of discrepancy were not consistent across sites, reliance on standardized adjustments to survey data using demographic factors may lead to substantial errors in estimation. These data suggest that programs must investigate and address setting-specific challenges to effectively address these challenges.

The disconnect between aggregate and individual-level agreement also demonstrates that validation based solely on comparing overall coverage percentages is insufficient. Programs need methods that assess individual-level concordance, such as spot-checks with household verification or systematic re-surveys of subsamples. WHO-recommended coverage evaluation and supervisory tools remain important for identifying broad implementation problems, but because they often rely on smaller samples and self-reported treatment status, they may not fully resolve the types of individual-level discrepancies observed here [2]. Treatment registers can provide reliable individual-level treatment status than self-reported surveys. However, this would require systematic, prospective, enumerated registers with a high level of quality control. In routine settings where registers may suffer from recording errors or incomplete documentation, their reliability remains uncertain. The survey timing of one to three weeks post-MDA represents a balance between minimizing recall bias and maintaining feasibility. The observed discrepancies in reporting suggest even this relatively short period may be subject to such bias or that other factors beyond recall period contribute substantially to measurement error. Future validation studies could examine whether concordance with treatment register records varies systematically across this window, for instance, comparing agreement at week one versus week three, which would provide empirical evidence on the magnitude of recall decay and help inform optimal survey timing recommendations for MDA programmes.

This study benefits from large sample sizes, multiple implementation rounds, diverse geographic settings, and rigorous data collection. Individual-level data linkage enabled direct concordance assessment, and site-stratified analysis revealed context-specific patterns that pooled analyses would obscure. Limitations include restriction to the final MDA round for consistency, although agreement patterns were broadly consistent across all rounds, suggesting this does not materially affect the conclusions. Additionally, kappa statistics should be interpreted with caution in this high-prevalence setting, as kappa values are sensitive to the marginal distribution of the outcome and may underestimate true agreement when prevalence is very high [14]. Treatment registers documented per-protocol delivery rather than directly observed ingestion, potentially introducing misclassification [13]. Covariates were limited to those available across all sites, and unmeasured factors such as literacy, health-seeking behaviour, or interviewer characteristics may be important determinants. Findings derive from a research trial and may not fully represent routine program settings. Because DeWorm3 treatment registers were prospectively maintained, census-linked, and supported by intensive field supervision, the observed direction and magnitude of discrepancy should be interpreted cautiously. In routine MDA programs, register completeness, denominator accuracy, and survey implementation may vary, and discrepancies between data sources may differ from those observed in this trial. Complete-case analysis excluded individuals with missing data from the primary regression models, although most of the round 6 analytic sample was retained. Education was excluded from the primary model because it was missing or not recorded for a substantial proportion of individuals; education-included sensitivity analyses were therefore based on a smaller and more selected sample and should be interpreted cautiously.

These findings suggest that MDA coverage surveys may systematically misclassify individual treatment status even when relatively rigorous. For programs operating near critical coverage thresholds, measurement error at observed levels may adversely impact programmatic interpretation, particularly if disagreement is directional or concentrated within specific subgroups. High aggregate coverage estimates do not accurately reflect individual-level measurement, suggesting a need for programs to consider more systematic high quality validation approaches. The WHO supervisor’s coverage tool offers practical mechanisms for identifying broad implementation gaps through spot-checks and household verification, but because they often rely on smaller samples and self-reported treatment status, they may not fully resolve the types of individual-level discrepancies observed in this study [2]. Linked survey-register validation, targeted register audits, and household re-checks in selected clusters may provide more actionable information on whether discrepancies reflect survey reporting error, register incompleteness, or true gaps in treatment delivery.

## Conclusions

Coverage measurement discrepancies between surveys and treatment registers are substantial, persistent, and context-specific. Improving measurement accuracy requires context-adapted approaches rather than standardized solutions. Programs should strengthen both treatment register quality and coverage survey implementation while recognizing that high aggregate estimates may mask important individual-level error. As a practical validation approach, WHO and national programme managers should consider linked survey-register checks in a sentinel subset of post-MDA coverage evaluations, particularly where reported coverage is close to programmatic thresholds or where survey and register estimates diverge. Further validation studies within routine MDA programs are needed to determine how coverage misclassification operates under real-world implementation conditions.

## Methods

### Ethical considerations

The DeWorm3 trial received ethical approval from the National Ethics Committee for Scientific Research (Benin), Christian Medical College, Vellore Institutional Review Board (India), College of Medicine Research Ethics Committee (COMREC) (Malawi), London School of Hygiene and Tropical Medicine, and University of Washington Institutional Review Board. The trial was registered at ClinicalTrials.gov NCT03014167 [5,7].

Written informed consent was obtained from all survey participants or guardians using translated consent forms in local languages. For treatment registers, consent was obtained during the census enumeration process [5].

### Study design and setting

This secondary analysis used data from the DeWorm3 trial, a cluster-randomized controlled trial designed to assess the feasibility of interrupting STH transmission through community-wide MDA as compared to school-based deworming. We sought to evaluate individual-level agreement between post-MDA coverage survey responses and trial-based treatment registers, and to identify factors associated with discrepant treatment classification. In DeWorm3, each country site in Benin, India, and Malawi consisted of one governmental administrative unit with a population of at least 80,000 individuals, which was divided into 40 geographically defined clusters, each with a minimum population of 1,650 individuals. These clusters were used for randomization, implementation, and sampling, and were randomized 1:1 to community-wide MDA or standard-of-care school-based deworming. Data was collected across six rounds of biannual drug distribution conducted between 2018 and 2020. Full details of the trial design, randomization procedures, data collection methods, and primary outcomes are reported in the trial protocol and primary outcome paper [5,7].

### Data sources

Prior to initiating the first MDA round, a comprehensive household census was conducted in all study clusters to enumerate the eligible population and collect demographic information including age, sex, education level, occupation, and household characteristics. GPS coordinates were recorded for each household to facilitate spatial analysis and future follow-up. The census was updated annually throughout the study period to account for births, deaths, migration, and other demographic changes. These census data served as the sampling frame for the coverage surveys and provided the denominators for calculating coverage rates, ensuring accurate population representation in the evaluation process [5,7].

Estimates of coverage were assessed using structured, digitized treatment registers developed for the purpose of the study [15]. At each individual household, these registers were completed by trained study field workers. These individuals accompanied Community Drug Distributors (CDDs) in Benin and India, and by Health Surveillance Assistants (HSAs) in Malawi, were responsible for delivering treatment to all eligible residents identified during census activities. Trained field workers accompanied them during distribution to ensure accurate recording of treatment status. This dual-personnel approach helped maintain data quality while facilitating community engagement through the CDDs’ and HSAs established relationships with community members [5,7].

Coverage surveys were conducted using multi-stage cluster sampling with 50 households randomly selected per cluster using probability proportional to size sampling, based on census data. Surveys were conducted between one and three weeks after completion of each round of MDA. In each household, adults were interviewed directly; for absent adults and for children (defined as individuals aged 1–15 years in Benin and India and 2–15 years in Malawi), information was provided by a household proxy or primary caregiver. If households could not be reached after three attempts on different days, replacements were selected from a pre-determined secondary list of 20 households per cluster. To minimise potential bias, field workers were rotated across clusters such that staff who conducted coverage surveys had not been involved in drug distribution in the same cluster.

### Study population and eligibility criteria

All individuals’ resident in the study area who were above one years of age in Benin and India and above two years of age in Malawi (per national guidelines) were included in the study population. Pregnant women in their first trimester and severely ill individuals were not eligible for treatment according to WHO guidelines [16]. Individuals who were not eligible for treatment (outside of target age range, died between the census and MDA, or pregnant in the first trimester) were not included in coverage denominators [5,11].

The primary analytic sample was defined in several steps. First, individuals with linked coverage survey and treatment register records were identified for round six across both trial arms. Second, the sample was restricted to the intervention arm because this analysis focused on community-wide MDA delivered through the DeWorm3 intervention. Third, individuals with missing treatment status in either data source were excluded from the concordance analysis. Round six was selected for the primary regression analysis to provide a consistent final-round analytic sample across sites. To assess whether agreement patterns were consistent over time, we also conducted descriptive concordance analyses for rounds 1–5. These descriptive round-specific analyses were based on separate matched intervention-arm samples within each MDA round rather than the fixed round six analytic sample; therefore, sample sizes varied by round, and individuals were not required to contribute observations to all six rounds.

### Outcomes

The primary outcome of this analysis was individual-level discrepancy between self-reported treatment status in the coverage survey and register-documented treatment delivery in the DeWorm3 treatment register. Discrepancies were examined in secondary analyses to descriptively characterize the direction of measurement error.

Treatment status from coverage surveys was defined based on self-reported swallowing of albendazole while treatment status from treatment registers was defined as a record of treatment delivery per-protocol, including instances where drugs were left at the household for individuals who were absent at the time of distribution [11]. Discrepancy captured any mismatch between coverage survey and treatment register status regardless of direction. Over-reporting represented instances where individuals reported treatment in the coverage survey but were recorded as untreated in the treatment register, indicating that survey data overestimated actual treatment. Under-reporting represented instances where individuals reported not being treated in the coverage survey but were recorded as treated in the treatment registers, indicating that survey data underestimated actual treatment. For each site and MDA round, discrepancy, over-reporting, and under-reporting percentages were calculated using the number of matched intervention-arm individuals with non-missing treatment status in both the coverage survey and treatment register as the denominator.

### Explanatory variables

After merging census, coverage survey and treatment log data using unique individual identifiers, demographic and contextual variables were obtained from the linked dataset. The variables include age (grouped as infants under one year, pre-school age children aged 1–4 years, school-age children 5–14 years, and adults 15 years and older), sex, education level (ranging from no formal education to higher education) and relative wealth (reported by quintile). Relative wealth (based on an wealth index) was calculated using Principal Components Analysis, taking the first principal component, from a list of household assets including house characteristics and appliance, technology, and livestock ownership (with additional fertilizer and water, sanitation and hygiene [WASH] access variables included in Malawi) [5,11].

Additional data regarding campaign awareness prior to implementation, information sources (family/friends, community health workers, radio, television, or printed materials), treatment location (home or outside their homes), and previous campaign participation were also included [11]. Geographic context was captured through residential classification as rural, peri-urban, or urban, based on population density and infrastructure assessments conducted during site characterization [5,11].

### Statistical analysis

Descriptive statistics summarized the distribution of treatment coverage, discrepancy, over-reporting, and under-reporting across the three study sites. Treatment coverage was calculated separately for coverage surveys (proportion of individuals reporting they swallowed albendazole) and treatment registers (proportion recorded as treated with albendazole). Agreement between the two measurement methods was assessed using percent discrepancy, defined as the proportion of matched individuals whose treatment status differed between data sources, and Cohen’s kappa statistics was reported descriptively.

To identify factors associated with discrepancies between coverage surveys and treatment registers, separate logistic regression models were fit for each study site using the round six intervention-arm analytic sample. The binary outcome was any discrepancy between survey-reported treatment status and register-documented treatment status. Models were stratified by site rather than pooled to account for anticipated contextual variations in implementation approaches, population characteristics, and health system factors that might influence reporting accuracy differently across settings. Each model included age (continuous, per 10-year increase), sex, MDA awareness, cluster type (rural vs periurban/urban), religion (majority vs minority), wealth quintile. Because education was missing or not recorded for a substantial proportion of individuals, particularly in Benin and Malawi, education was not included in the primary regression model. Complete-case analysis was applied to the covariates included in the primary models, excluding individuals with missing data on any covariate.

As sensitivity analyses, we repeated the site-specific models including education as a categorical variable among individuals with defined education data, including an unknown/missing education category where available. Education-related findings were interpreted cautiously because the education-included models retained a substantially smaller and more selected analytic sample. Covariates showing no within-site variation after restriction to complete cases were excluded from site-specific models to avoid estimation problems. Odds ratios with 95% confidence intervals were reported for all retained covariates, with statistical significance assessed at the α = 0.05 level.

Data management and security procedures followed institutional protocols as described in primary trial publications [11]. All analyses were conducted using R version 4.5.0 (R Foundation for Statistical Computing, Vienna, Austria).

## Supporting information

S1 Table Comparison of individuals included versus excluded from the round 6 intervention-arm analytic sample. (DOCX)

S2 Table Sensitivity analysis including education. (DOCX)

S1 Fig Sample flow for analytic sample. (TIF)
