# Supplementary material for: Individual-level agreement between coverage surveys and treatment registers in mass drug administration: A secondary analysis of the multi-country DeWorm3 cluster randomized trial
Source: PLoS Negl Trop Dis. 2026 Sep 21;20(9):e0014740. doi: 10.1371/journal.pntd.0014740 (PMC13619018; doi:10.1371/journal.pntd.0014740)
Supplement: Supplementary file 1 [file pntd.0014740.s001.docx]

**S1 Table . Comparison of individuals included versus excluded from the round 6 intervention-arm analytic sample.**

| **Characteristic** | **Overall**^1^ | **Included**^1^ | **Excluded**^1^ | **p-value**^2^ |
| --- | --- | --- | --- | --- |
| **Site** |  |  |  | <0.001 |
| Benin | 8,464 (34%) | 4,069 (33%) | 4,395 (35%) |  |
| India | 7,223 (29%) | 3,747 (31%) | 3,476 (28%) |  |
| Malawi | 9,064 (37%) | 4,346 (36%) | 4,718 (37%) |  |
| **Trial arm** |  |  |  | 0.5 |
| Control | 2 (<0.1%) | 0 (0%) | 2 (<0.1%) |  |
| Intervention | 24,749 (100%) | 12,162 (100%) | 12,587 (100%) |  |
| **Reason for exclusion** |  |  |  | <0.001 |
| Included | 12,162 (49%) | 12,162 (100%) | 0 (0%) |  |
| Missing treatment status in survey or register | 12,587 (51%) | 0 (0%) | 12,587 (100%) |  |
| Not in intervention arm or missing arm | 2 (<0.1%) | 0 (0%) | 2 (<0.1%) |  |
| **Age category** |  |  |  | <0.001 |
| ≤1 year | 146 (0.6%) | 0 (0%) | 146 (1.2%) |  |
| 1–4 years | 2,490 (10%) | 1,095 (9.0%) | 1,395 (11%) |  |
| 5–14 years | 6,389 (26%) | 3,349 (28%) | 3,040 (24%) |  |
| ≥15 years | 15,716 (63%) | 7,717 (63%) | 7,999 (64%) |  |
| Unknown | 10 (<0.1%) | 1 (<0.1%) | 9 (<0.1%) |  |
| **Sex** |  |  |  | 0.089 |
| Male | 11,845 (48%) | 5,753 (47%) | 6,092 (48%) |  |
| Female | 12,906 (52%) | 6,409 (53%) | 6,497 (52%) |  |
| Unknown | 0 (0%) | 0 (0%) | 0 (0%) |  |
| **Religion** |  |  |  | <0.001 |
| Hinduism | 6,933 (28%) | 3,635 (30%) | 3,298 (26%) |  |
| Christianity | 5,920 (24%) | 2,837 (23%) | 3,083 (24%) |  |
| Islam/Muslim | 8,972 (36%) | 4,294 (35%) | 4,678 (37%) |  |
| None | 370 (1.5%) | 191 (1.6%) | 179 (1.4%) |  |
| Voodoo | 2,169 (8.8%) | 1,036 (8.5%) | 1,133 (9.0%) |  |
| Other | 119 (0.5%) | 64 (0.5%) | 55 (0.4%) |  |
| Unknown | 268 (1.1%) | 105 (0.9%) | 163 (1.3%) |  |
| **Education** |  |  |  | <0.001 |
| No education | 5,832 (24%) | 3,006 (25%) | 2,826 (22%) |  |
| Any Primary education | 3,286 (13%) | 1,564 (13%) | 1,722 (14%) |  |
| Any Middle education | 855 (3.5%) | 451 (3.7%) | 404 (3.2%) |  |
| Any Secondary education | 1,903 (7.7%) | 963 (7.9%) | 940 (7.5%) |  |
| Any Higher secondary education | 697 (2.8%) | 309 (2.5%) | 388 (3.1%) |  |
| College/Diploma/University | 782 (3.2%) | 379 (3.1%) | 403 (3.2%) |  |
| Unknown | 11,396 (46%) | 5,490 (45%) | 5,906 (47%) |  |
| **Marital status** |  |  |  | <0.001 |
| Unmarried | 3,445 (14%) | 1,547 (13%) | 1,898 (15%) |  |
| Married monogamous | 8,354 (34%) | 4,269 (35%) | 4,085 (32%) |  |
| Married polygamous | 398 (1.6%) | 201 (1.7%) | 197 (1.6%) |  |
| Separated | 376 (1.5%) | 187 (1.5%) | 189 (1.5%) |  |
| Divorced (Legally) | 77 (0.3%) | 34 (0.3%) | 43 (0.3%) |  |
| Widow/Widower | 963 (3.9%) | 486 (4.0%) | 477 (3.8%) |  |
| Unknown | 11,138 (45%) | 5,438 (45%) | 5,700 (45%) |  |
| **Wealth index quintile** |  |  |  | <0.001 |
| Lowest quintile | 4,012 (16%) | 2,086 (17%) | 1,926 (15%) |  |
| Low quintile | 4,519 (18%) | 2,163 (18%) | 2,356 (19%) |  |
| Medium quintile | 4,852 (20%) | 2,435 (20%) | 2,417 (19%) |  |
| High quintile | 5,326 (22%) | 2,580 (21%) | 2,746 (22%) |  |
| Highest quintile | 6,026 (24%) | 2,895 (24%) | 3,131 (25%) |  |
| Unknown | 16 (<0.1%) | 3 (<0.1%) | 13 (0.1%) |  |
| **Cluster type** |  |  |  | 0.3 |
| Rural | 16,112 (65%) | 7,955 (65%) | 8,157 (65%) |  |
| Periurban/Urban | 8,639 (35%) | 4,207 (35%) | 4,432 (35%) |  |
| Unknown | 0 (0%) | 0 (0%) | 0 (0%) |  |
| **MDA awareness** |  |  |  | <0.001 |
| Not aware | 3,868 (16%) | 3,834 (32%) | 34 (0.3%) |  |
| Aware | 7,589 (31%) | 7,540 (62%) | 49 (0.4%) |  |
| Unknown | 13,294 (54%) | 788 (6.5%) | 12,506 (99%) |  |
| **Coverage survey treatment status** |  |  |  | <0.001 |
| Reported treated in survey | 12,088 (49%) | 12,043 (99%) | 45 (0.4%) |  |
| Reported not treated in survey | 146 (0.6%) | 119 (1.0%) | 27 (0.2%) |  |
| Missing | 12,517 (51%) | 0 (0%) | 12,517 (99%) |  |
| **Treatment register status** |  |  |  | <0.001 |
| Recorded treated in register | 21,373 (86%) | 11,732 (96%) | 9,641 (77%) |  |
| Recorded not treated in register | 2,763 (11%) | 430 (3.5%) | 2,333 (19%) |  |
| Missing | 615 (2.5%) | 0 (0%) | 615 (4.9%) |  |
| ^1^n (%) | | | | |
| ^2^Pearson's Chi-squared test | | | | |
