# Supplementary material for: Individual-level agreement between coverage surveys and treatment registers in mass drug administration: A secondary analysis of the multi-country DeWorm3 cluster randomized trial
Source: PLoS Negl Trop Dis. 2026 Sep 21;20(9):e0014740. doi: 10.1371/journal.pntd.0014740 (PMC13619018; doi:10.1371/journal.pntd.0014740)
Supplement: Supplementary file 2 [file pntd.0014740.s002.docx]

S2 Table. Sensitivity analysis including education

|  | **Benin (n=1,966)** | | | **India (n=2,306)** | | | **Malawi (n=1,787)** | | |
| --- | --- | --- | --- | --- | --- | --- | --- | --- | --- |
| **Covariate** | **OR** | **95% CI** | **p-value** | **OR** | **95% CI** | **p-value** | **OR** | **95% CI** | **p-value** |
| **Age (per 10-year increase)** | 0.89 | 0.77, 1.02 | 0.11 | 1.10 | 0.87, 1.37 | 0.4 | 0.81 | 0.69, 0.94 | 0.01 |
| **Sex** |  |  |  |  |  |  |  |  |  |
| Female (ref) | 1.0 | 1.0 |  | 1.0 | 1.0 |  | 1.0 | 1.0 |  |
| Male | 0.77 | 0.51, 1.15 | 0.2 | 0.80 | 0.42, 1.50 | 0.5 | 0.46 | 0.29, 0.71 | <0.001 |
| **MDA aware** |  |  |  |  |  |  |  |  |  |
| Not aware (ref) | 1.0 | 1.0 |  | 1.0 | 1.0 |  | 1.0 | 1.0 |  |
| Aware | 0.89 | 0.56, 1.47 | 0.6 | 0.81 | 0.44, 1.52 | 0.5 | 0.72 | 0.47, 1.11 | 0.14 |
| **Cluster type** |  |  |  |  |  |  |  |  |  |
| Rural (ref) | 1.0 | 1.0 |  | 1.0 | 1.0 |  | 1.0 | 1.0 |  |
| Periurban/Urban | 3.53 | 2.12, 6.11 | <0.001 | 1.18 | 0.55, 2.34 | 0.7 | 1.55 | 0.97, 2.43 | 0.06 |
| **Minority religion** |  |  |  |  |  |  |  |  |  |
| Majority religion (ref) | 1.0 | 1.0 |  | 1.0 | 1.0 |  | 1.0 | 1.0 |  |
| Minority religion | 0.87 | 0.54, 1.38 | 0.6 | 0.65 | 0.04, 3.09 | 0.7 | 1.43 | 0.63, 3.00 | 0.4 |
| **Wealth index quintile** |  |  |  |  |  |  |  |  |  |
| Lowest quintile (ref) | 1.0 | 1.0 |  | 1.0 | 1.0 |  | 1.0 | 1.0 |  |
| Low quintile | 1.15 | 0.58, 2.29 | 0.7 | 0.65 | 0.16, 2.51 | 0.5 | 0.62 | 0.30, 1.25 | 0.2 |
| Medium quintile | 0.68 | 0.34, 1.36 | 0.3 | 0.96 | 0.28, 3.51 | >0.9 | 0.36 | 0.15, 0.78 | 0.01 |
| High quintile | 0.74 | 0.38, 1.45 | 0.4 | 2.05 | 0.71, 6.80 | 0.2 | 0.65 | 0.33, 1.28 | 0.2 |
| Highest quintile | 0.56 | 0.29, 1.13 | 0.10 | 1.33 | 0.42, 4.71 | 0.6 | 0.74 | 0.38, 1.45 | 0.4 |
| **Education** |  |  |  |  |  |  |  |  |  |
| 1 (ref) | 1.0 | 1.0 |  | 1.0 | 1.0 |  | 1.0 | 1.0 |  |
| 2 | 0.99 | 0.53, 1.82 | >0.9 | 0.48 | 0.03, 2.49 | 0.5 | 1.25 | 0.70, 2.29 | 0.5 |
| 3 | 1.02 | 0.24, 3.06 | >0.9 | 0.94 | 0.35, 2.43 | >0.9 | 0.59 | 0.03, 3.08 | 0.6 |
| 4 | 0.76 | 0.39, 1.44 | 0.4 | 1.02 | 0.15, 4.11 | >0.9 | 2.63 | 1.18, 5.75 | 0.02 |
| 5 | 0.87 | 0.20, 2.66 | 0.8 | 0.93 | 0.24, 2.92 | >0.9 | 0.67 | 0.10, 2.64 | 0.6 |
| 6 | 0.86 | 0.47, 1.58 | 0.6 | 2.30 | 0.90, 6.07 | 0.086 | 1.65 | 0.08, 12.0 | 0.7 |
| 7 | 1.81 | 0.91, 3.48 | 0.08 | 0.00 |  | >0.9 | 2.47 | 1.23, 4.96 | 0.01 |
| *Abbreviations: CI = Confidence Interval, OR = Odds Ratio. Education categories represent adult educational attainment: 1 = no formal education; 2–3 = primary education; 4–5 = secondary education; 6 = post-secondary education; 7 = unknown or missing education. Category 1 (no formal education) was used as the reference group.* | | | | | | | | | |
